# Hydrogen peroxide dynamics in subcellular compartments of malaria parasites using genetically encoded redox probes

**DOI:** 10.1038/s41598-017-10093-8

**Published:** 2017-09-05

**Authors:** Mahsa Rahbari, Stefan Rahlfs, Jude M. Przyborski, Anna Katharina Schuh, Nicholas H. Hunt, David A. Fidock, Georges E. Grau, Katja Becker

**Affiliations:** 1 Biochemistry and Molecular Biology, Interdisciplinary Research Center, Justus Liebig University Giessen, 35392 Giessen, Germany; 2 Department of Parasitology, Philipps University Marburg, 35043 Marburg, Germany; 3 Discipline of Pathology, Sydney Medical School and Bosch Institute, University of Sydney, Medical Foundation Building (K25), Camperdown, NSW 2050 Australia; 4 Department of Microbiology and Immunology and Division of Infectious Diseases, Department of Medicine, Columbia University Medical Center, New York, 10032 USA

## Abstract

Redox balance is essential for the survival, growth and multiplication of malaria parasites and oxidative stress is involved in the mechanism of action of many antimalarial drugs. Hydrogen peroxide (H_2_O_2_) plays an important role in redox signalling and pathogen-host cell interactions. For monitoring intra- and subcellular redox events, highly sensitive and specific probes are required. Here, we stably expressed the ratiometric H_2_O_2_ redox sensor roGFP2-Orp1 in the cytosol and the mitochondria of *Plasmodium falciparum (P. falciparum)* NF54-*attB* blood-stage parasites and evaluated its sensitivity towards oxidative stress, selected antimalarial drugs, and novel lead compounds. In both compartments, the sensor showed reproducible sensitivity towards H_2_O_2_ in the low micromolar range and towards antimalarial compounds at pharmacologically relevant concentrations. Upon short-term exposure (4 h), artemisinin derivatives, quinine and mefloquine impacted H_2_O_2_ levels in mitochondria, whereas chloroquine and a glucose-6-phosphate dehydrogenase (G6PD) inhibitor affected the cytosol; 24 h exposure to arylmethylamino steroids and G6PD inhibitors revealed oxidation of mitochondria and cytosol, respectively. Genomic integration of an H_2_O_2_ sensor expressed in subcellular compartments of *P. falciparum* provides the basis for studying complex parasite-host cell interactions or drug effects with spatio-temporal resolution while preserving cell integrity, and sets the stage for high-throughput approaches to identify antimalarial agents perturbing redox equilibrium.

## Introduction

Despite strong worldwide efforts to combat malaria, the spread of parasites resistant to effective and affordable antimalarials including chloroquine (CQ) has worsened the situation (reviewed in ref. 1). The World Health Organization (WHO) recommends artemisinin-based combination therapies (ACTs) for the treatment of uncomplicated malaria caused by *P. falciparum* ^2^. However, resistance has emerged to artemisinin (ART) and its derivatives as well as their ACT partner drugs^2^. The development of novel antimalarial agents that are efficacious against multidrug-resistant parasites (reviewed in ref. 3) therefore has high priority. One pathway to novel drug discovery is to exploit the susceptibility of the parasite to imbalances in its redox equilibrium^4^. *P. falciparum* is exposed to substantial oxidative stress during its intraerythrocytic life cycle, in part due to the oxidative burst of macrophages activated by the host immune system during malaria infection^5, 6^. Internal production of oxidants derived from the electron transport chain may also occur^7^. In addition, degradation of haemoglobin within its acidic digestive vacuole (DV) leads to the generation of H_2_O_2_^8^, the most important reactive oxygen species (ROS) in cells. Long regarded as a toxic byproduct that can damage macromolecules including DNA, proteins and lipids^9^, H_2_O_2_ is increasingly recognized as an important cellular signalling molecule with regulatory functions^10, 11^. H_2_O_2_ selectively oxidizes reactive cysteine residues and thereby controls functions of redox-sensitive proteins^12, 13^. The antioxidant defence of *P. falciparum* comprises the thiol-based glutathione (GSH) and thioredoxin (Trx) systems (reviewed in ref. 14) as well as a range of peroxiredoxins^15^, and superoxide dismutases. Notably, malaria parasites lack a genuine catalase and glutathione peroxidase^16^, which supports the notion that the redox system of *P. falciparum* is an attractive drug target. Many antimalarial drugs exert their activity, at least partially, through the generation of oxidative stress^17–20^. However, there is little known about specific molecular targets within the parasite that mediate its susceptibility to oxidative challenge. Current methods for detecting redox changes in malaria parasites and trypanosomes, such as biochemical assays and fluorescent dyes, have recently been reviewed^21^. However, besides some serious drawbacks and the risks of artefacts, none of the approaches used to date can be applied to specifically quantify ROS in subcellular compartments of cultured malaria parasites.

Recently, we reported on the transient expression of the genetically encoded fluorescent H_2_O_2_ redox probes roGFP2-Orp1 and HyPer-3 in the cytosol of *P. falciparum*, which paved the way for non-disruptive, ratiometric, real-time, and dynamic measurements of H_2_O_2_ levels^22^. Both sensors were found to be highly selective and sensitive in detecting submicromolar concentrations of H_2_O_2_^22^. However, the roGFP2-Orp1 sensor was found to be more suitable for complex studies in *Plasmodium* since the necessity to use a pH probe in parallel made utilizing HyPer-3 more challenging and time consuming. RoGFP2-Orp1 consists of a redox-sensitive green fluorescent protein (roGFP2) fused to a highly sensitive thiol peroxidase (Orp1). H_2_O_2_-induced conformational changes of the probe can be detected in cells via confocal laser scanning microscopy (CLSM) after excitation at 405 and 488 nm, with emission set at 510 nm^23^. Exposure to H_2_O_2_ leads to oxidation being mediated from H_2_O_2_ via Orp1 to roGFP2, resulting in an almost stoichiometric oxidation of the probe^23^. When roGFP2 is oxidized, the excitation peak at 405 nm increases, while the 488 nm peak decreases. By calculating the ratio of 405/488 nm, the extent of oxidation can be determined.

Here, we report on the stable genomic integration of the roGFP2-Orp1 probe into blood stages of the CQ-sensitive *P. falciparum* strain NF54-*attB*. The sensor was targeted to the cytosol and, for the first time, to a subcellular compartment of malaria parasites, the mitochondrion, by using the *attB*×*attP* system of integrative recombination. The mycobacteriophage Bxb1 serine integrase mediates site-specific recombination between the phage *attP* site of the pDC2 expression plasmid and an integrated *attB* site in *P. falciparum* ^24, 25^. Stable integration of the sensor reduces the heterogeneous fluorescence signals that can arise in episomal expression systems^22^. Using this integrated reporter line, we were able to systematically analyze by CLSM the effects of oxidative and pharmacological stress on the cytosolic and mitochondrial H_2_O_2_ metabolism of *P. falciparum*. Results, presented below, demonstrate that the stably integrated roGFP2-Orp1 sensor provides a robust tool for differentially studying H_2_O_2_ homeostasis in subcellular compartments of *P. falciparum*.

## Results

### Expression of stably integrated roGFP2-Orp1 and Mito-roGFP2-Orp1 sensors in P. falciparum NF54-attB parasites

**Figure 1:**
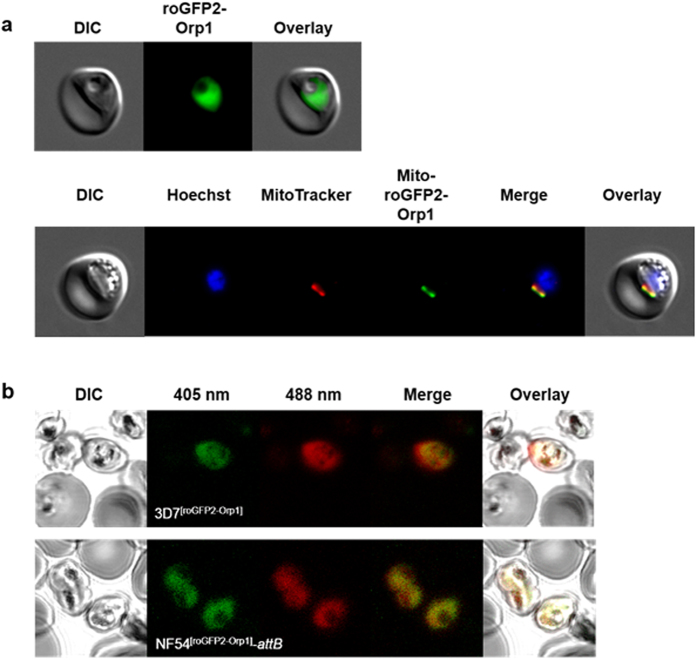
Confirmation of cytosolic and mitochondrial localization of the roGFP2-Orp1 probe in *P. falciparum* NF54^[roGFP2-Orp1]^-*attB* and NF54^[Mito-roGFP2-Orp1]^-*attB* parasites, respectively, and comparison of the expression rates of 3D7^[roGFP2-Orp1]^ and NF54^[roGFP2-Orp1]^-*attB* parasites. (**a**) Fluorescence microscopy of *P. falciparum* NF54^[roGFP2-Orp1]^-*attB* parasites shows cytosolic localization of the probe, whereas NF54^[Mito-roGFP2-Orp1]^-*attB* parasites show mitochondrial localization of the probe carrying the target sequence of CS. (**b**) In *P. falciparum* 3D7^[roGFP2-Orp1]^ parasites approx. 60% of the parasites expressed the redox sensor roGFP2-Orp1^22^, whereas in NF54^[roGFP2-Orp1]^-*attB* cells the expression rate was 100%.

The roGFP2-Orp1 and Mito-roGFP2-Orp1 redox sensors were cloned into the stable NF54-*attB* strain under the control of the *calmodulin* promoter using the pDC2-CAM-[X]-bsd-*attP* plasmid^24^. Parasites were detectable in cell culture three weeks after stable transfection. 100% of the parasites expressed the H_2_O_2_ probes. The redox probes roGFP2-Orp1 and Mito-roGFP2-Orp1 were successfully targeted to the cytosol and the mitochondrion of *P. falciparum* NF54-*attB*, respectively. For targeting the mitochondrion the signal sequence of citrate synthase (CS) was fused to roGFP2-Orp1. The presence of the probes in transfected *P. falciparum* NF54-*attB* trophozoites was analyzed by fluorescence microscopy. Mitochondrial localization of the probe was confirmed by using MitoTracker Orange. The nucleus was stained with Hoechst 33258 (Fig. 1a). In episomally transfected *P. falciparum* 3D7^[roGFP2-Orp1]^ cells, fluorescence signals differed considerably between single cells^22^, whereas in the stably transfected NF54^[roGFP2-Orp1]^-*attB*, all parasites showed homogenous fluorescence intensities (Fig. 1b). Parasite viability was not negatively affected by the cytosolic or the mitochondrial sensor as deduced from identical generation time and morphology. The parental NF54-*attB* line showed a multiplication rate of 6–7, the sensor targeted parasites a multiplication rate of 5–6.

### NF54^[roGFP2-Orp1]^-attB and NF54^[Mito-roGFP2-Orp1]^-attB parasites exhibit similar H_2_O_2_ sensitivities

**Figure 2:**
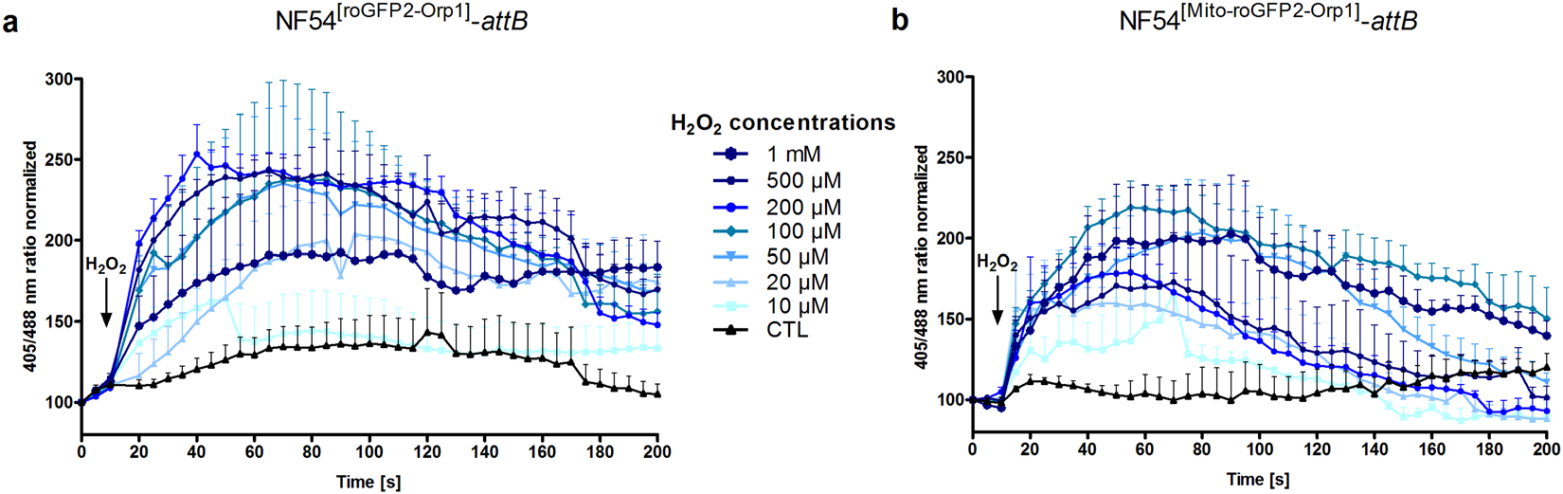
Effect of H_2_O_2_ on *P. falciparum* NF54^[roGFP2-Orp1]^-*attB*- and NF54^[Mito-roGFP2-Orp1]^-*attB* -transfected parasites_._ After 15 s of baseline monitoring, NF54^[roGFP2-Orp1]^-*attB* (**a**) and NF54^[Mito-roGFP2-Orp1]^-*attB* parasites (**b**) were exposed to H_2_O_2_ (10 µM to 1 mM) and were monitored for 3 min via CLSM. Non-treated parasites served as controls. NF54^[roGFP2-Orp1]^-*attB* and NF54^[Mito-roGFP2-Orp1]^-*attB* parasites showed comparable sensitivities towards H_2_O_2_. For each H_2_O_2_ concentration, data from at least nine trophozoites in total, examined in three independent experiments, were analyzed per data point. Means and standard errors of the means (SEM) of three experiments are shown.

In order to measure the effects of H_2_O_2_ on the redox probes in the parasites, enriched NF54^[roGFP2-Orp1]^-*attB* (Fig. 2a) and NF54^[Mito-roGFP2-Orp1]^-*attB* trophozoites (Fig. 2b) were exposed to H_2_O_2_ concentrations ranging from 10 µM to 1 mM and were monitored for 3 min via CLSM. For live-cell imaging we chose only parasites that showed fluorescent signals at both 405 and 488 nm excitation and resided within an intact host cell (≥99% of the cells fulfilled these requirements). The probes were adjusted with 10 mM dithiothreitol (DTT) to the fully reduced or with 1 mM diamide (DIA) to the fully oxidized states (each using 2 min incubations). To better compare the functionality of the two sensors the obtained ratio values of a time course were all normalized to the first basal ratio value, which was set to 100. To monitor potential effects of the excitation light on the cellular redox state, we imaged cells for 3 min under the same experimental conditions but without treatment. As determined by confocal laser scanning microscopy (CLSM), both probes were only present either in the parasites’ cytosol (roGFP2-Orp1) or mitochondrion (Mito-roGFP2-Orp1), thereby confirming successful targeting of these probes to specific subcellular compartments. Upon H_2_O_2_ exposure, both NF54^[roGFP2-Orp1]^-*attB* and NF54^[Mito-roGFP2-Orp1]^-*attB* transfectants showed a comparable concentration-dependent increase in the 405/488 nm ratio, in which as little as 10 µM H_2_O_2_ led to an increase in fluorescence ratio (1.5-fold) (Fig. 2a,b). At 20 µM, H_2_O_2_ seemed to directly oxidize the mitochondrial probe (1.6-fold), which was followed by the reduction of H_2_O_2_ (Fig. 2b), whereas the cytosolic probe was oxidized more slowly but almost twice as much (Fig. 2a). The addition of 50 µM and 100 µM H_2_O_2_ increased the 405/488 nm ratio by 2.0 to 2.3-fold for both sensors. Parasites expressing roGFP2-Orp1 were maximally oxidized (up to 2.5-fold) with 200 µM and 500 µM H_2_O_2_, and a 1.8-fold increase in fluorescence ratio could be detected for the mitochondrial sensor. Exposure to 1 mM H_2_O_2_ led to a similar increase in 405/488 nm ratio for NF54^[Mito-roGFP2-Orp1]^-*attB* transfectants (2.0-fold) compared with NF54^[roGFP2-Orp1]^-*attB* parasites (1.9-fold), with oxidation remaining rather constant over time. NF54^[Mito-roGFP2-Orp1]^-*attB* parasites exhibited a control (CTL) that remained constant over time (Fig. 2b) in comparison to NF54^[roGFP2-Orp1]^-*attB* parasites, which showed a 1.3-fold increase in the 405/488 nm ratio over time (Fig. 2a). NF54^[Mito-roGFP2-Orp1]^-*attB* showed faster rates of H_2_O_2_ reduction as compared with NF54^[roGFP2-Orp1]^-*attB*. However, after short-term treatment with high H_2_O_2_ concentrations (100 µM and higher), leakage of the roGFP2-Orp1 sensor into the erythrocyte cytosol became visible (see Supplementary Video S1 of NF54^[roGFP2-Orp1]^-*attB* parasites).

### High-resolution imaging of NF54^[roGFP2-Orp1]^-Z attB trophozoites

The response of NF54^[roGFP2-Orp1]^-*attB* trophozoites to H_2_O_2_ concentrations of ≥20 µM was further studied via high-resolution 3D-imaging using a Leica SP8 Confocal Fluorescence Microscope (see Supplementary Video S1 using 500 µM H_2_O_2_). The first 10 s of the video shows a steady-state image of the first frame of the time series indicating the basal redox state of the parasites. After 13 s (≙ video 00:45 min), 500 µM H_2_O_2_ was added to the parasites and the effects on the green and red fluorescence signals became visible. After 17 s (≙ video 01:45 min), we observed a fully oxidized roGFP2-Orp1 sensor gradually leaking into the host cell. From 37 s (≙ video 06:45 min) onwards we present a steady-state image of the last frame of the time series.

### Determination of direct interaction of antimalarial drugs with recombinant roGFP2-Orp1 in vitro

*In vitro* measurements with the redox sensor roGFP2-Orp1 were performed to differentiate between pharmacological effects of antimalarial drugs and direct interactions with the probe. We tested the clinically used drugs amodiaquine (AQ), atovaquone (ATQ), lumefantrine (LUM), primaquine (PQ), and the experimental agents rotenone (Rot) (an electron transport chain blocker) and 2-deoxyglucose (2-DG) (a glycolysis inhibitor). In addition, we investigated the effects of two novel antimalarial lead compounds, namely the arylmethylamino steroid 1o^26^ and the G6PD inhibitor ML304^27^. All compounds were used at concentrations of 10 nM to 1 mM in standard reaction buffer. The effects of the clinically used drugs artemisinin (ART), artemether (ATM), artesunate (ATS), chloroquine (CQ), quinine (QN), and mefloquine (MQ) on recombinant roGFP2-Orp1 had been described previously^22^ and did not affect the fluorescence ratio at the concentrations used in this study. Supplementary Table S1 summarizes the effects of the above-mentioned compounds on the 405/480 nm fluorescence ratios of recombinant roGFP2-Orp1 at 100 µM and 1 mM, as determined in a plate reader after 0 min, 5 min, 4 h, and 24 h incubation at 25 °C. As shown, the tested compounds did not have an effect on the fluorescence ratio even at the highest concentrations tested. The maximal concentrations used in this study were in the lower micromolar range.

### The redox probes roGFP2-Orp1 and Mito-roGFP2-Orp1 have similar dynamic ranges in living parasites

**Figure 3:**
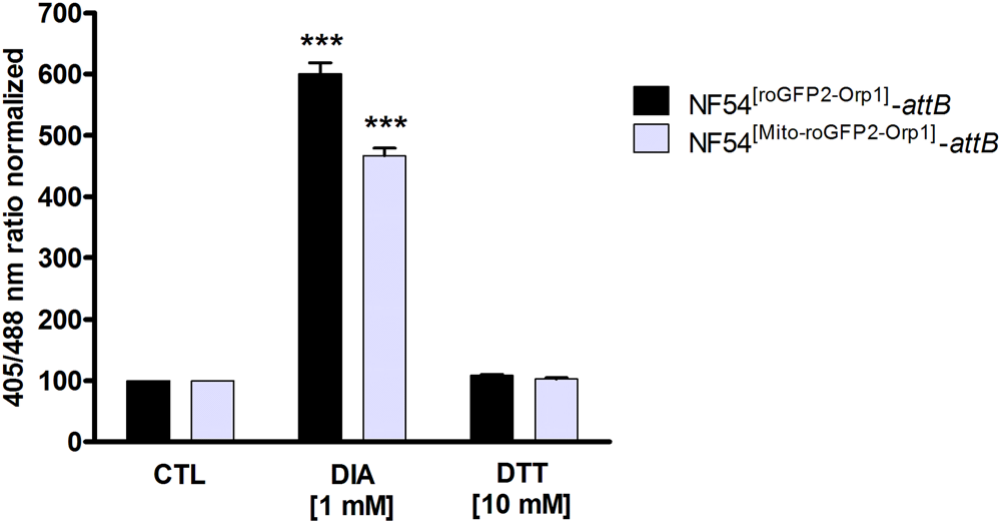
Dynamic range of roGFP2-Orp1 and Mito-roGFP2-Orp1 in transfected NF54-*attB* parasites. NF54-*attB* parasites transfected with roGFP2-Orp1 or Mito-roGFP2-Orp1 were exposed to 1 mM DIA or 10 mM DTT for 2 min before blocking with 2 mM NEM. Fluorescence ratios of 405/488 nm were detected with CLSM. NF54^[roGFP2-Orp1]^-*attB* parasites showed a slightly higher DIA sensitivity than NF54^[Mito-roGFP2-Orp1]^-*attB* parasites. CLSM data were composed of values from at least 10–20 trophozoites analyzed per experiment. Mean values and standard errors of the means (±SEM) are shown for three independent experiments. A one-way ANOVA test with 95% confidence intervals with the Dunnett’s Multiple Comparison Test was applied for statistical analysis of significance (***p < 0.001).

To measure the completely oxidized or reduced state of the probes, NF54^[roGFP2-Orp1]^-*attB*- and NF54^[Mito-roGFP2-Orp1]^-*attB*-enriched trophozoites were incubated for 2 min with 1 mM DIA or 10 mM DTT, respectively, blocked thereafter with 2 mM N-ethylmaleimide (NEM), and monitored via CLSM (Fig. 3). To calculate the dynamic range of the two redox probes in the parasites, the fluorescence ratio of fully oxidized NF54^[roGFP2-Orp1]^-*attB*- and NF54^[Mito-roGFP2-Orp1]^-*attB* was divided by the fluorescence ratio of the fully reduced state. As shown in Fig. 3, treatment with 1 mM DIA led to a significant increase of the fluorescence ratio in both NF54^[roGFP2-Orp1]^-*attB*- and NF54^[Mito-roGFP2-Orp1]^-*attB* transfectants (Fig. 3). NF54^[roGFP2-Orp1]^-*attB* showed a dynamic range of 5.5 and NF54^[Mito-roGFP2-Orp1]^-*attB* a slightly lower dynamic range of 4.5.

### Effects of pharmacologically active compounds on NF54^[roGFP2-Orp1]^-attB and NF54^[Mito-roGFP2-Orp1]^-attB parasites

**Figure 4:**
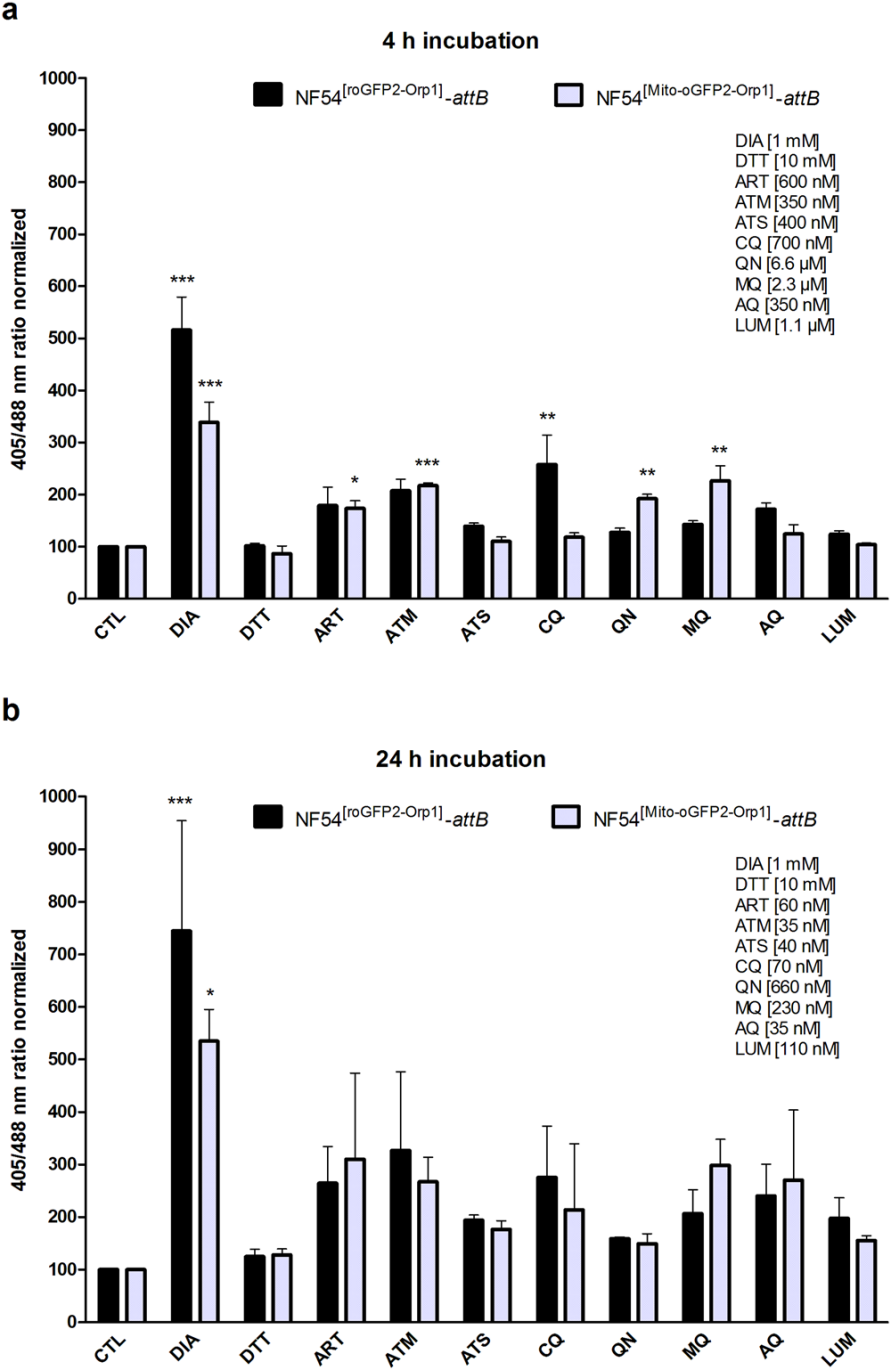
Mid- and long-term effects of ART, ATM, ATS, CQ, QN, MQ, AQ, and LUM on the redox ratio of *P. falciparum* NF54^[roGFP2-Orp1]^-*attB* and NF54^[Mito-roGFP2-Orp1]^-*attB* transfected parasites. 4 h incubation of NF54^[roGFP2-Orp1]^-*attB* transfectants with CQ led to a significant increase of fluorescence ratio as detected using CLSM. (**a**) NF54^[Mito-roGFP2-Orp1]^ transfectants were significantly oxidized by ART, ATM, QN and MQ. (**b**) 24 h incubations oxidized both probes but did not lead to a significant increase of the 405/488 nm ratio. CLSM data were obtained from 10 to 20 trophozoites for each experiment and each incubation time. Mean values and standard errors of the means (±SEM) are shown for three independent experiments. A one-way ANOVA test with 95% confidence intervals with the Dunnett’s Multiple Comparison Test was applied for statistical analysis of significance (*p < 0.05; **p < 0.01; ***p < 0.001).

To determine whether selected antimalarial drugs and redox-active agents can directly affect H_2_O_2_ levels in the cytosol and mitochondrion of *P. falciparum* asexual blood stages, we carried out mid- and long-term experiments with NF54^[roGFP2-Orp1]^-*attB*- and NF54^[Mito-roGFP2-Orp1]^-*attB*-transfected parasites assayed using CLSM. To measure the completely oxidized and reduced state of the probes, magnetically-enriched trophozoites were incubated for 2 min with 1 mM DIA or 10 mM DTT respectively, and blocked thereafter with 2 mM NEM. Control studies excluded a direct interaction of the chosen drugs with the H_2_O_2_ probes. For mid-term experiments (4 h), we incubated 26–30 h post-invasion trophozoites with 100 × EC_50_ of ART, ATM, ATS, CQ, QN, MQ, AQ, or LUM, and subsequently blocked reactions with 2 mM NEM. EC_50_ values for the NF54-*attB* strain were determined using SYBR Green assays^28^ and are shown in Supplementary Table S2. Concentrations used for these experiments can be found as Supplementary Table S3. Due to fluctuations in the basal fluorescence ratios of individual parasites, we normalized the obtained ratio values to the control value that was set to 100. Both ART and ATM increased the 405/488 nm ratio of the mitochondrial probe significantly (p < 0.05 and p < 0.001, respectively), as did QN (p < 0.01) and MQ (p < 0.01) (Fig. 4a). Only CQ showed a significant effect on the cytosolic probe (p < 0.01). For long-term drug exposure experiments (24 h), ring-stage parasites were incubated with 10 × EC_50_ of the following drugs: ART, ATM, ATS, CQ, QN, MQ, AQ, and LUM (see Supplementary Table S2). Prior to magnetic enrichment, cysteines were blocked with 2 mM NEM. Oxidation of both the cytosolic and mitochondrial probes in the range of 2.0 to 3.0-fold of the basal 405/488 nm ratio was observed upon incubation with all drugs, although experimental variability was quite large as shown by the larger error bars and these changes failed to reach statistical significance (Fig. 4b). Interestingly, 24 h incubation with ATM led to a partially cytosolic fluorescence signal of the Mito-roGFP2-Orp1 sensor in both the 405 nm and 488 nm channels.

### Effects of Rot, 2-DG, ATQ, PQ, compound 1o, and ML304 on the oxidation levels of NF54^[roGFP2-Orp1]^-attB- and NF54^[Mito-roGFP2-Orp1]^-attB-transfected parasites

**Figure 5:**
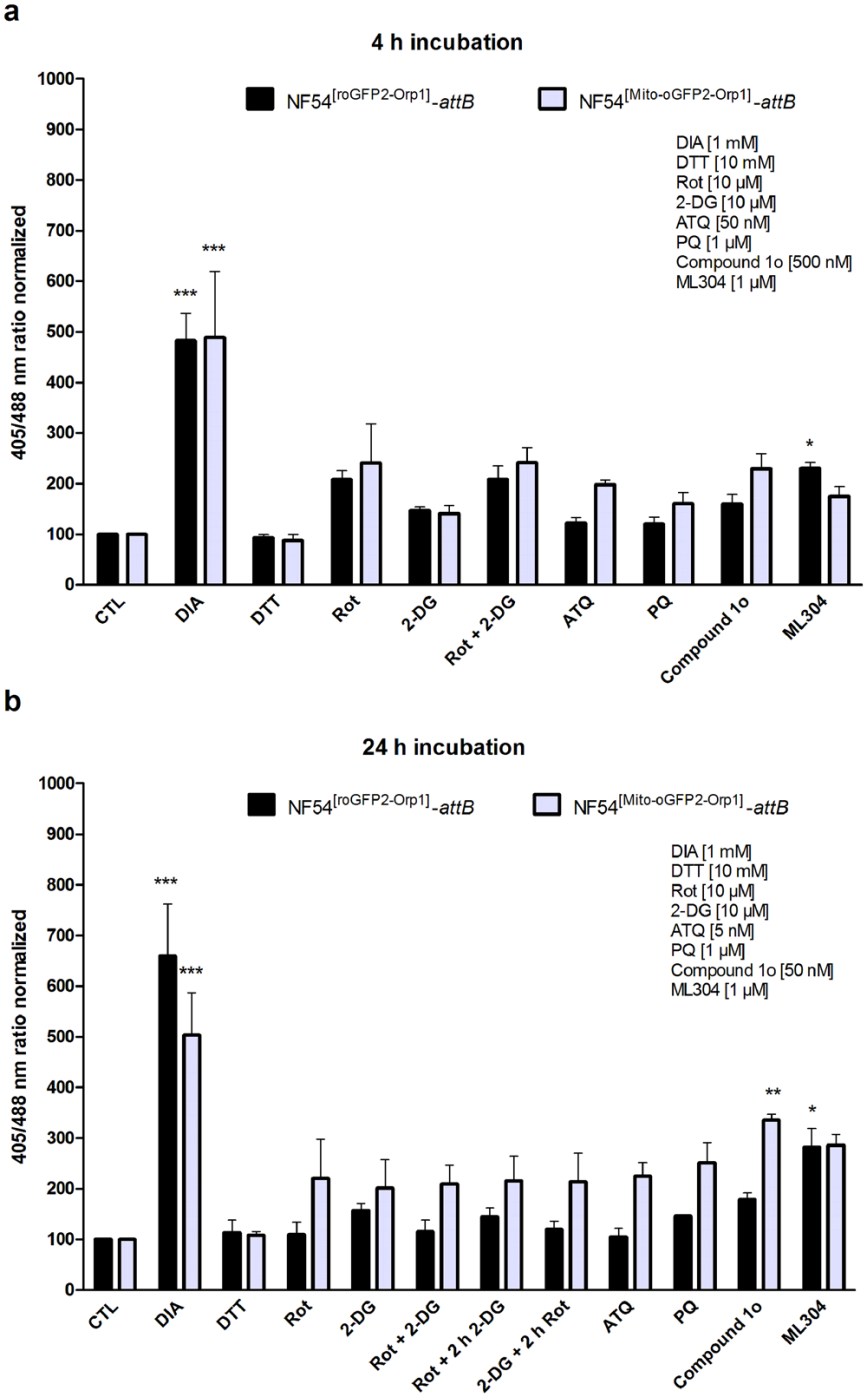
Mid- and long-term effects of Rot, 2-DG, ATQ, PQ, compound 1o and ML304 on the redox ratio of *P. falciparum* NF54^[roGFP2-Orp1]^-*attB* and NF54^[Mito-roGFP2-Orp1]^-*attB*-transfected parasites. (**a**) 4 h incubation of NF54^[roGFP2-Orp1]^-*attB* transfectants with the compounds led to an increase of fluorescence ratio for both sensors as determined using CLSM. ML304 in particular significantly increased the 405/488 nm ratio of roGFP2-Orp1. (**b**) 24 h incubations with the drugs seemed to have a higher influence on the fluorescence ratio for NF54^[Mito-roGFP2-Orp1]^-*attB* transfectants, in which compound 1o significantly increased the mitochondrial probe signal. NF54^[roGFP2-Orp1]^-*attB* transfectants were significantly oxidized by ML304. CLSM data were composed of 10–20 trophozoites analyzed per experiment for each incubation. Mean values and standard errors of the means (±SEM) are shown for three independent experiments. A one-way ANOVA test with 95% confidence intervals with the Dunnett’s Multiple Comparison Test was applied for statistical analysis of significance (*p < 0.05; **p < 0.01; ***p < 0.001).

Mid- and long-term exposures were carried out with NF54^[roGFP2-Orp1]^-*attB*- and NF54^[Mito-roGFP2-Orp1]^-*attB*-transfected parasites via CLSM as described above. Control studies excluded a direct interaction of the selected drugs with the purified H_2_O_2_ probes. For mid-term experiments (4 h) 26–30 h trophozoites were incubated with 100 × EC_50_ or fixed values of Rot, 2-DG, ATQ, PQ, compound 1o, and ML304. Reactions were subsequently blocked with 2 mM NEM. EC_50_ values for the NF54-*attB* strain are shown in Supplementary Table S2. Concentrations used for the experiments can be found as Supplementary Table S3. Only ML304 significantly oxidized NF54^[roGFP2-Orp1]^-*attB* parasites (p < 0.05) (Fig. 5a). For long-term drug exposure experiments (24 h), ring-stage parasites were incubated with 10 × EC_50_ or fixed concentrations of the compounds Rot, 2-DG, ATQ, PQ, compound 1o, and ML304 (see Supplementary Table S2). Prior to enrichment, cysteines were blocked with 2 mM NEM. Compound 1o had a significant effect on the 405/488 nm ratio of Mito-roGFP2-Orp1 (p < 0.01) and ML304 significantly increased the 405/488 nm ratio of cytosolic roGFP2-Orp1 (p < 0.05) (Fig. 5b).

## Discussion

H_2_O_2_ plays a crucial role in signalling cascades that regulate numerous cellular functions. Genetically encoded fluorescent probes are unique tools to study H_2_O_2_ production in living cells of different scales and complexities. These sensors offer high specificity, reversibility, subcellular targeting and transgenic options in the native environment without harming the cells and tissues. In this study, we applied live cell imaging with the cytosolically and mitochondrially expressed H_2_O_2_ biosensor roGFP2-Orp1 and Mito-roGFP2-Orp1, respectively, in asexual blood stages of stably transfected *P. falciparum* NF54-*attB* parasites. The results provided suggest that the mode of action of some antimalarial drugs and some of the novel antimalarial compounds includes disturbance of H_2_O_2_ homeostasis within the malaria parasite.

In line with our previous study using transiently expressed roGFP2-Orp1 in *P. falciparum* ^22^, we observed a rapid, dynamic, and ratiometric response of roGFP2-Orp1 and Mito-roGFP2-Orp1 upon oxidation with H_2_O_2_ (Fig. 2). The dynamic ranges of roGFP2-Orp1 and Mito-roGFP2-Orp1 in cells in oxidizing vs. reducing states (DIA/DTT) were determined to be 5.5 and 4.5, respectively (Fig. 3), which is comparable to earlier reports of 5 and 6 in *P. falciparum* and *Drosophila* respectively^22, 29^.

We note that NF54^[roGFP2-Orp1]^-*attB* transfectants were susceptible to phototoxicity, as confirmed via short-term time courses presented as CTL (Fig. 2). In contrast, the fluorescence ratio of NF54^[Mito-roGFP2-Orp1]^-*attB* transfectants remained rather constant over time. Phototoxicity is one of the major problems in live-cell imaging, occurring upon illumination of fluorescently labelled cells^30^. This leads to the production of free radicals^30^ and ROS^31^ that damage cells. The susceptibility of the H_2_O_2_ sensors roGFP2-Orp1 and Mito-roGFP2-Orp1 to H_2_O_2_ might explain why *P. falciparum* NF54-*attB* parasites expressing these sensors react to light-induced stress, as shown by an increased 405/488 nm ratio. We also note that the mitochondrial inner membrane is permeable to H_2_O_2_, as the externally added oxidant was able to induce immediate changes in 405/488 nm fluorescence ratio (Fig. 2b). Overall, both sensors responded to different H_2_O_2_ concentrations in their own specific ways (Fig. 2a,b). The oxidation of the roGFP2-Orp1 probe is reversible in *P. falciparum*, as shown after DIA exposure by adding DTT that decreased the ratio to the baseline redox state by reducing the disulfide bonds^22^. Our high-resolution video (Supplementary Video S1) illustrates for the first time the leakage phenomenon of the roGFP2-Orp1 sensor into the host cell upon addition of 500 µM H_2_O_2_.

Targeting the roGFP2-Orp1 sensor to the mitochondrion paves the way for measuring dynamic redox changes induced by H_2_O_2_ in living *Plasmodium* parasites in discrete subcellular compartments, while preserving cell integrity. This is an advantage over other currently available imaging agents such as Amplex Red^32^ or CM-H_2_DCFDA^33^, which are prone to artefacts and preclude compartment-specific monitoring of H_2_O_2_ dynamics (reviewed in ref. 21).

The oxidizing effects of the antimalarial drugs ART, ATM, ATS, CQ, QN, MQ, AQ, LUM depicted in Fig. 4 (some representing trends, while others reached levels of significance) appeared to be more pronounced (although with higher variability between single cells) after 24 h of incubation (10 × EC_50_) than after 4 h (100 × EC_50_), indicating that a long exposure time to a drug increases its impact on the H_2_O_2_ level. ART and ATM led to significant increases in 405/488 nm fluorescence ratios of the mitochondrial Mito-roGFP2-Orp1 probe after 4 h incubation (p < 0.05 and p < 0.001, respectively) (Fig. 4a). Longer (24 h) incubations of these drugs led to more pronounced increases in the ratio but these did not reach significance. The antimalarial effect of ART and its derivatives is mediated by the endoperoxide moiety that interacts with reduced haem generated from haemoglobin degradation^34^ leading to radical formation. The generated radicals damage parasite cellular macromolecules, including proteins and lipids^35, 36^. The mode of action mainly responsible for the antiparasitic effect of ART is still not fully understood due to its several molecular targets in a cell, e.g. DNA synthesis, glycolysis, haemoglobin digestion^35^, and mitochondrial membrane potential (∆_Ψm_)^37^. Mutations in the propeller domain of the kelch protein K13 of *P. falciparum* confer ART resistance, and K13 function has been putatively linked to oxidative stress^38–43^. In the current study, after 24 h incubation with ATM, a partially cytosolic expression of the mitochondrial probe was observed in the 405 nm and 488 nm channels, indicating putative disruption of the mitochondria and leakage of the probe into the cytosol of the parasite. This phenomenon recently was also observed with the mitochondrial glutathione probe Mito-roGFP2-hGrx1 and ART in *P. falciparum* 3D7 parasites^44^ (Franziska Mohring, pers. comm.). These data are consistent with an earlier report of leakage of the cytosolic probe roGFP2-Orp1 in *P. falciparum* 3D7 transfectants into the host cell upon exposure to high H_2_O_2_ concentrations (100 µM to 1 mM)^22^.

Among the quinolines tested (CQ, QN, MQ, and AQ), only CQ significantly increased the ratio of roGFP2-Orp1 after a 4 h treatment (p < 0.01), whereas a significant ratio increase of Mito-roGFP2-Orp1 was detected with QN and MQ (both p < 0.01) (Fig. 4a). Quinolines are thought to inhibit haemoglobin degradation within the DV of the parasite by acting as ferriprotoporphyrin detoxification inhibitors. Formation of nontoxic crystalline haemozoin is reduced^14, 45^. The CQ-haem complex is toxic for the parasite, whereas GSH complex formation with haem in the DV prevents oxidative damage^46^. CQ-resistant parasites have multiple mutations in the CQ resistance transporter PfCRT, an integral membrane protein in the DV^47^. Mutant PfCRT isoforms have been proposed to exhibit glutathione transport activity, with increased transport of GSH into the DV in CQ-resistant parasites^14, 48, 49^. LUM is an antimalarial agent used to treat acute uncomplicated malaria. It is administered in combination with ATM for improved efficacy^50^. Incubation with LUM for 4 h barely affected the 405/488 nm ratio of both cytosolic and mitochondrial probes, comparable to the effects of ATS on these redox probes (Fig. 4a). However, 24 h incubation led to an increase in fluorescence ratio of roGFP2-Orp1 and Mito-roGFP2-Orp1 (Fig. 4b).

Rot is a plant toxin and blocks NAD^+^ linked oxidation in complex I of the ETC. Blocking ETCs is known to increase the production of ROS, e.g. H_2_O_2_ in the mitochondrion^51, 52^. Reducing equivalents are necessary for the detoxification of H_2_O_2_, and are provided by glucose via the formation of pyruvate^53, 54^ as well as through the regeneration of NADPH^55–57^. Inhibitors of the glycolysis pathway have shown potent antimalarial activity, including 2-DG, which significantly reduced *P. falciparum* parasite survival^58, 59^ and rapidly depleted parasite ATP^58, 60^. Our results suggest that Rot and 2-DG could increase intracellular H_2_O_2_ levels, however, the effects failed to be statistically significant (Fig. 5a,b).

ATQ is a coenzyme Q analogue that specifically targets the cytochrome bc1 complex of the mitochondrial respiratory chain in the malarial parasite^61^. This inhibition leads to the collapse of the mitochondrial membrane potential^62^. PQ’s mode of action is still poorly understood but mitochondrial disruption^63^ and increased oxidative stress^64^ have been implicated. For ATQ and PQ, our results showed increased fluorescence ratios of Mito-roGFP2-Orp1 in both 4 h and 24 h incubations, consistent with their putative mechanisms of action of targeting the mitochondria and increasing oxidative stress.

Compound 1o is a recently discovered lead steroid that was shown to be fast acting and highly potent against *P. falciparum* blood stages of CQ-sensitive and resistant parasites (EC_50_ 1–5 nM)^26^. Potent activity was also observed with gametocytes for all stages^26^. The steroid and the hydroxyarylmethylamino moieties were essential for antimalarial activity, which indicated a chelate-based quinone methide mechanism involving metal or haem bioactivation. Oral administration of compound 1o drastically reduced parasitaemia in *P. berghei-*infected mice and cured the animals^26^. Furthermore, parasite transmission from mice to mosquitoes was efficiently blocked by compound 1o^26^. In studies employing the cytosolic glutathione redox sensor hGrx1-roGFP2, incubation with nanomolar concentrations of compound 1o led to a dose-dependent increase of the redox ratio, indicating oxidation and alterations in the intracellular redox potential^26^. In the present study, compound 1o increased both cytosolic and mitochondrial H_2_O_2_ levels in 4 h and 24 h incubation experiments, with a significant effect on Mito-roGFP2-Orp1 after 24 h. These findings suggest a putative impact of compound 1o on mitochondria metabolism leading to increasing H_2_O_2_ levels, a phenomenon that merits further analysis.

G6PD is a novel target for antimalarial drug design, based on observations that G6PD deficiency in humans protects the host from severe malaria infections^65^. G6PD catalyses the first step in the pentose phosphate pathway, a key metabolic pathway yielding NADPH, an essential reducing equivalent in the antioxidative defence of cells^66^. A lack of G6PD leads to a lack of reducing equivalents, an increase in oxidative stress and, thus, to host protection against severe malaria. NADPH in parasite-infected red blood cells (iRBCs) is generated by human G6PD, but also by *P. falciparum* glucose-6-phosphate dehydrogenase 6-phosphogluconolactonase (*Pf*GluPho) with G6PD activity. *Pf*GluPho is essential for *Plasmodium* proliferation and propagation and differs structurally and mechanistically from the human ortholog^67, 68^. Therefore, developing *Pf*GluPho inhibitors is a promising way to kill the parasite and treat malaria. ML304 is a ring-expanded chemical scaffold and selectively inhibits *Pf*GluPho (EC_50_ < 1 µM), but not human G6PD (>420-fold selectivity)^69^. It is the second reported *Pf*G6PDH inhibitor following ML276^27^. In the present study, inhibiting cytosolic *Pf*GluPho led to a significant increase in fluorescence ratio of the cytosolic roGFP2-Orp1 sensor in 4 h and 24 h experiments (Fig. 5a,b). ML304 affected the fluorescence ratio of the mitochondrial probe in 4 h experiments and showed a more pronounced effect in 24 h incubations.

Taken together, all drugs tested in our study showed evidence of effects (with clear trends often reaching significance) on H_2_O_2_ homeostasis either in the cytosol, the mitochondrion or both compartments, in *P. falciparum* NF54-*attB* parasites exposed to drugs for 4 h and 24 h incubation periods. Therefore, increased H_2_O_2_ levels might play a role in their mode of action in parasites. However, it also should be considered that some of the tested drugs do not directly act at the cytosol or mitochondrion level but instead are more likely to act at other sites such as the DV that might account for the non-significant effects. Beside the already known and pharmacologically used antimalarial drugs such as artemisinins and quinolines, the novel steroid compound 1o and the *Pf*GluPho inhibitor ML304 were found to significantly induce oxidative stress in malaria parasites by elevating intracellular H_2_O_2_ levels.

Our results present the first significant step in stably expressing genetically encoded redox sensors in *Plasmodium* via genomic integration, with fluorescence signals in 100% of the parasites. Our stable transfection method overcomes issues of transient transfection such as the low percentage of sensor expression, the occurrence of parasites resistant to the selective drug, and the observed loss of fluorescence intensity over time. The H_2_O_2_ redox probes roGFP2-Orp1 and Mito-roGFP2-Orp1 proved to be sensitive and reliable tools for studying the H_2_O_2_ redox milieu in the cytosol and the mitochondrion of intracellular parasites. This genomic integration of redox sensors enables the application of high-throughput screening of bulk cell culture parasites after drug exposure as well as more complex in-cell studies using a plate-reader based approach as an alternative to single live-cell imaging.

## Materials and Methods

### Drugs and chemicals

All chemicals used were of the highest available purity and were obtained from Roth (Karlsruhe, Germany), Sigma-Aldrich (Steinheim, Germany), or Merck (Darmstadt, Germany). RPMI 1640 medium was from Gibco (Paisley, United Kingdom). ART was from Roth, CQ, ATS, AQ and ATQ from Sigma-Aldrich (Steinheim, Germany), MQ from Roche (Mannheim, Germany), ATM from TCI Germany (Eschborn), AQ and ATQ from Sigma-Aldrich, and QN from Acros Organics (Geel, Belgium). LUM was kindly provided by the Novartis Institute of Tropical Diseases to David Fidock (Columbia University, New York, USA), compound 1o by Dr. Reimar Krieg, Jena, Institute of Anatomy II, University Hospital Jena, Germany, and ML304 by Dr. Anthony Pinkerton, Conrad Prebys Center for Chemical Genomics, Sanford-Burnham-Prebys Medical Discovery Institute, La Jolla, CA. Rot was obtained from Roth and 2-DG from Sigma. WR99210 was kindly supplied by Jacobus Pharmaceuticals, New Jersey, USA. Stock solutions of DIA, DTT, CQ, and 2-DG were dissolved in sterile distilled H_2_O, while ART, ATM, ATS, AQ, QN, MQ, ATQ, LUM, compound 1o, and ML304 were dissolved in DMSO.

### Cloning the roGFPF2-Orp1 and Mito-roGFP2-Orp1 constructs

The sensor roGFP2-Orp1 recently was transiently expressed in 3D7 *P. falciparum* parasites^22^ by using the pARL1a(+) expression vector and modified for stable integration in this study. Mito-roGFP2-Orp1 previously was generated by introducing first KpnI restriction sites at the mitochondrial signal sequence of citrate synthase (CS) that was inserted at the *N*-terminus of the roGFP2-Orp1 construct. For in-cell experiments with the NF54-*attB* strain, roGFP2-Orp1 and Mito-roGFP2-Orp1 were cloned into the pDC2-CAM-*attP* expression vector with the CAM 5′ promoter using AvrII and XhoI restriction sites. All primers used are listed in the supporting information section (see Supplementary Table S3). Confirmation of successful integration of the plasmid was investigated via PCR and agarose gel electrophoresis (see Supplementary Table S4). Heterologous overexpression of roGFP2-Orp1 for the evaluation of the *in vitro* interactions of drugs and redox-active compounds with the recombinant redox probe was performed according to ref. 22.

### In vitro characterization of recombinant roGFP2-Orp1

All drugs and compounds, DIA, DTT, and H_2_O_2_ were diluted either with a standard reaction buffer (100 mM potassium phosphate, 1 mM EDTA, pH 7.0) or buffer with 50% v/v DMSO to a final concentration of 1 mM and equal concentrations of DMSO in every well, and used immediately. DIA [1 mM] and DTT [10 mM] served as controls in all experiments for identifying maximal oxidation and reduction, respectively. Prior to the experiments, the reaction buffer was degassed by ultrasound for 1 h at RT. Purified recombinant roGFP2-Orp1 protein was reduced with 20 mM DTT for 30 min at 4 °C, desalinated (Zeba^TM^ Spin Desalting Columns, Thermo Scientific), and diluted in reaction buffer to a final concentration of 5 µM. A 5-fold drug/redox-active compound dilution (10 µl) was mixed with 40 µl of 5 µM roGFP2-Orp1 in a 96-well microplate (black, half-area, Greiner Bio-One, Frickenhausen). Prior to fluorescence measurements via plate reader (Clariostar, BMG Labtech), protein concentration and loading time were optimized. The ratios of the fluorescence signals at 405/475 nm were calculated for roGFP2-Orp1 (emission at 510 nm), and plotted against time or concentration of antimalarial drugs/redox-active compounds. Data from two to three independent experiments were analyzed for each concentration (see Supplementary Table S1).

### P. falciparum cell culture

The chloroquine (CQ)-sensitive NF54-*attB* of *P. falciparum* was cultured as described^22^. Briefly, the strain was propagated in RBCs (A+) in RPMI 1640 medium supplemented with 0.5% w/v Albumax, 9 mM glucose, 0.2 mM hypoxanthine, 2.1 mM L-glutamine, 25 mM Hepes, and 22 µg/ml gentamycin at 3.3% haematocrit and 37 °C in a gaseous mixture consisting of 3% O_2_, 3% CO_2_, and 94% N_2_. Synchronization of *P. falciparum* parasites was carried out with 5% (w/v) sorbitol^70^. *P. falciparum* trophozoites were enriched via magnetic separation^71^. Cell lysates were obtained via saponin lysis^72^. Parasitaemia was counted on Giemsa-stained blood smears.

### Transfection of P. falciparum NF54-attB parasites

Trophozoite-stage parasites were transferred to fresh RBCs (≤7 days) after magnet enrichment (5% haematocrit, 10 ml culture). 5 ml of NF54-*attB* culture (ring-stage 8–10 h, 5–8% parasitaemia) were centrifuged at 1,500 rpm for 3 min and the supernatant was discarded. For each transfection 50 µg of purified DNA with the target gene and 50 µg of purified pINT DNA were mixed and put on ice. The parasite pellet was resuspended with an equal volume of sterile cytomix and spun down at 1,500 rpm for 3 min. Excess cytomix was discarded and the volume remaining was brought up with extra cytomix, so that the total volume with DNA was approximately 450 µl. The culture was resuspended and was added to the DNA (on ice). The parasites were electroporated in an electroporation cuvette (2 mm) (310 V, 950 µF, capacitance ∞) with the Bio-Rad Gene Pulser^73^. The time constant was between 10 and 15 ms. The electroporated parasites were immediately resuspended with 1 ml complete medium and transferred to a 15 ml conical containing 3.5 ml complete medium and RBCs (4% haematocrit). The parasites were spun down at 1,500 rpm for 3 min, resuspended in new complete medium (5 ml) and plated out. The parasites were placed into the incubator for at least 1 h until the medium was removed and replaced with fresh complete medium. After 24 h the selection drugs blasticidin [2.5 µg/ml] and gentamycin (G418) [125 µg/ml] were added to the cells to select for transfectants for six days. Culture medium was changed every day for the first six days and thereafter every other day. On day six post-electroporation, 50 µl of fresh RBC were added to the cells and from day seven onwards only blasticidin was used for selection. Between day 10 and 13 the cells were cut 1:2 every week until the appearance of parasites (usually three to four weeks). After confirmation of the stable integration of the DNA of interest via PCR, agarose gel electrophoresis and fluorescence microscopy, the selection drug blasticidin was omitted for the maintenance of the culture.

### Preparation of cloning plates

Cloning plates were prepared to obtain a homogenous parasite culture expressing the respective construct. Limiting dilution was employed for cloning with 0.5 iRBC/well and 0.25 iRBC/well in 96-well microplates (clear half-area, Greiner Bio-One, Frickenhausen) in the presence of uninfected erythrocytes. These platings were done with 0.2 ml/well corresponding to 2.5 iRBC/ml and 1.25 iRBC/ml. Cloning was initiated with predominantly ring-stage parasites (2–5% parasitaemia) and fresh RBC (≤7 days). All dilutions were made with complete medium containing RBC at 1.8% haematocrit. At day 7, 150 µL medium was removed without disturbing the cell monolayer and replaced with 200 µl fresh complete medium containing 0.4% fresh RBCs. At day 14, the same procedure as on day 7 was repeated. On day 20, detection of parasitized wells was prepared using a SYBR Green assay^28^. To each well of a separate black 96-well microplate plate (half-area, Greiner Bio-One, Frickenhausen) 15 µl of 3 × SYBR Green I nucleic acid gel stain (10,000 × stock solution) in lysis buffer (20 mM Tris-HCl, 5 mM EDTA, 0.16% w/v saponin, and 1.6% v/v Triton X-100) were added and 30 µl of the cloning plate cultures were mixed with the fluorescence dye. The plates were incubated for 30 min at 37 °C and fluorescence was measured (485 nm excitation filter, 535 nm emission filter) with a plate reader (Infinite M200, Tecan). Blood PCR was performed using the primers for stable integration to confirm the genomic integration of the constructs via agarose gel electrophoresis. The parasites were scaled up and experiments were performed on clones with the best fluorescence signals.

### In-cell localization studies of the sensor Mito-roGFP2-Orp1

For mitochondrial localization studies, the NF54^[Mito-roGFP2-Orp1]^-*attB* parasites were incubated with MitoTracker Orange 1:100,000 for 10–15 min in RPMI and Hoechst 33258 1:50,000 and then washed once with RPMI. Images were taken with a Zeiss Epi-fluorescence microscope. Images were acquired using appropriate filter sets on a Carl Zeiss Axio Observer inverse epifluorescence microscope, and processed with Fiji^74^.

### P. falciparum NF54-attB drug susceptibility assays

For calculating the half maximal effective concentration (EC_50_) of antimalarial drugs or compounds against *P. falciparum* NF54-*attB* parasites the SYBR Green I-based fluorescence assay was performed according to^28^ in 96-well format with modifications. Serial double dilutions (50 µl) of the compounds in complete medium were performed in 96-well half area microtiter plates (µClear bottom). Synchronized ring-stage parasites (50 µl) were added to each well (0.15% parasitaemia, 1.25% final haematocrit) and incubated for 48 h at 37 °C. To each well 20 μl of 5 × SYBR Green (10,000 × stock solution) in lysis buffer (20 mM Tris-HCl, 5 mM EDTA, 0.16% w/v saponin, and 1.6% v/v Triton X-100) were added and incubated for 24 h at RT in the dark. The fluorescence was measured with the Clariostar plate reader at ex 494 nm/em 530 nm. Curve-fitting the percentage growth inhibition against log drug concentration with a variable slope sigmoidal function allowed the determination of EC_50_ values (see Supplementary Table S2).

### Effects of H_2_O_2_ on the redox homeostasis of P. falciparum NF54-attB parasites

H_2_O_2_ concentrations of 10 µM, 20 µM, 50 µM, 100 µM, 200 µM, 500 µM, and 1 mM were added to NF54^[roGFP2-Orp1]^-*attB* and NF54^[Mito-roGFP2-Orp1]^-*attB*-transfected parasites in short-term (3 min) time course experiments. Trophozoite-stage parasites (26–30 h, 6–8% parasitemia) were magnetically enriched (Miltenyi Biotec, Germany), counted using a Neubauer haemocytometer (Brand GmbH, Germany), and returned to cell culture for at least 1 h to recover. The cells were washed once with pre-warmed Ringer’s solution (122.5 mM NaCl, 5.4 mM KCl, 1.2 mM CaCl_2_, 0.8 mM MgCl_2_, 11 mM D-glucose, 25 mM Hepes, 1 mM NaH_2_PO_4_, pH 7.4) and resuspended in Ringer’s solution at a final parasite concentration of 2.0 × 10^4^ trophozoites/µl. For every H_2_O_2_ concentration, at least three parasites from three independent experiments were analyzed per data point. All experiments included non-treated, fully reduced, and fully oxidized parasites as controls. For dynamic range time courses with DIA and DTT, data from three independent experiments of at least three parasites each were analyzed per data point. Mean and standard error of the mean (SEM) are shown. A one-way ANOVA test with 95% confidence intervals with the Dunnett’s Multiple Comparison Test (GraphPad Prism 5.0) was applied for statistical analysis of significance (*p < 0.05; **p < 0.01; ***p < 0.001).

For high resolution imaging NF54^[roGFP2-Orp1]^-*attB* trophozoites (26–30 h) were exposed to different concentrations of H_2_O_2_ and the fluorescence signals were dynamically monitored using z-stack at the Leica SP8 Confocal Fluorescence Microscope (Sydney University, Australia). Deconvolution, 3D visualization and movie making were performed by using the deconvolution software Huygens (Scientific Volume Imaging) and the image processing software Fiji^74^.

### Effects of redox-active compounds and antimalarial drugs on redox homeostasis

The half-maximal effective concentration (EC_50_) of drugs on *P. falciparum* NF54-*attB* asexual blood stages was determined with the SYBR Green assay (see Supplementary Table S2). The effects of antimalarial drugs and redox-active compounds on *P. falciparum* were investigated in mid- (4 h) and long-term (24 h) incubation experiments. For 4 h experiments, trophozoite-stage parasites (26–30 h) of NF54^[roGFP2-Orp1]^-*attB* and NF54^[Mito-roGFP2-Orp1]^-*attB* (6–8% parasitaemia) were magnetically enriched (Miltenyi Biotec, Germany), counted by using a Neubauer haemocytometer (Brand GmbH, Germany), and returned to cell culture (at 2.0 × 10^5^ trophozoites/µl) for at least 1 h to recover. 1.0 × 10^6^ cells in 100 µl cell culture medium were placed into LoBind tubes (Eppendorf) for 4 h incubation experiments. The parasites were treated with antimalarial drugs and redox-active compounds at 100 × EC_50_ for 4 h under cell culture conditions. Subsequently, free thiol groups were blocked with 2 mM NEM for 15 min at 37 °C and cells were resuspended in Ringer’s solution. For 24 h experiments, a 5 ml culture (5% haematocrit, 6–8% parasitaemia) of ring-stage parasites (6–10 h post invasion) was treated with antimalarial drugs and redox-active compounds at 10 × EC_50_ for 24 h. Prior to enrichment, cysteines were blocked with 2 mM NEM for 15 min at 37 °C. After incubation, cells were resuspended in Ringer’s solution. All experiments included non-treated parasites as control, and fully reduced and fully oxidized parasites with 10 mM DTT and 1 mM DIA, respectively (2 min incubation) prior to blocking with NEM. Each experiment was carried out three times. Between 10–20 microscopy images were taken each time. Mean and standard error of the mean (SEM) are shown. A one-way ANOVA test with 95% confidence intervals with the Dunnett’s Multiple Comparison Test (GraphPad Prism 5.0) was applied for statistical analysis of significance (**p < 0.05; **p < 0.01; ***p < 0.001).

### Confocal live-cell imaging and image processing

Magnetically enriched *P. falciparum* NF54*-attB* trophozoites (26–30 h) were resuspended with pre-warmed (37 °C) Ringer’s solution (122.5 mM NaCl, 5.4 mM KCl, 1.2 mM CaCl_2_, 0.8 mM MgCl_2_, 11 mM D-glucose, 25 mM Hepes, 1 mM NaH_2_PO_4_, pH 7.4), and seeded onto poly-L-lysine-coated µ-slides VI (for time course experiments) or µ-Slides 18 Well (flat) (Ibidi, Martinsried, Germany) for endpoint measurements. A Leica confocal system TCS SP5 inverted microscope equipped with the objective HCX PL APO 63.0 × 1.30 GLYC 37 °C UV connected to a 37 °C temperature chamber was used. The argon laser power was set to 20%; scanning was performed at 400 Hz frequency and at a 512 × 512 pixel resolution. The smart gain and smart offset were 950 V and −0.9%, respectively. The probes were excited at 405 nm and at 488 nm with a sequential scan, and emission was detected at 500–550 nm. Laser intensity for both lines, NF54^[roGFP2-Orp1]^-*attB* and NF54^[Mito-roGFP2-Orp1]^-*attB* was adjusted to match the full dynamic range of the probes to the dynamic range of the detector (roGFP2-Orp1: 405 nm: 10%, 488 nm: 4%; Mito-roGFP2-Orp1: 405 nm: 10%, 488 nm: 5%).

For time series, images were acquired every 5 s over a time course of approximately 3 min after 15 s of basal measurements. Autofluorescence images were simultaneously taken at ex 405 nm/em 430–450 nm and individually defined together with the background for every image but no fluorescence signal could be detected. The Leica LAS AF Lite software for fluorescence analysis was used. The 405/488 nm ratios were calculated. The graphs were plotted using the GraphPad Prism 5 software (San Diego, CA, USA). Only parasites showing fluorescent signals at both 405 and 488 nm excitation and an intact host cell were chosen. In order to investigate the short-term effects of DIA and DTT (dynamic range measurements) on NF54^[roGFP2-Orp1]^-*attB* and NF54^[Mito-roGFP2-Orp1]^-*attB* infected parasites, 50 µl of cells (1.0 × 10^6^ trophozoites) were exposed to 1 mM of DIA or 10 mM of DTT, and the fluorescence signals were monitored in a time course of approximately 3 min. Due to the fluctuations of the basal 405/488 nm ratio of single parasites, the obtained ratio values of a time course were all related to the first basal ratio value, which was set to 100. All experiments were performed three times and included non-treated parasites as controls, and endpoint experiments both fully reduced and fully oxidized parasites (2 min incubation prior to measurements). For each time course at least three parasites were assessed per experiment. Endpoint experiments comprised between 10–20 parasites per experiment. Data are representative of three independent experiments.

### Data availability

All data generated or analyzed during this study are included in this published article (and its Supplementary Information files).

## Electronic supplementary material

Supplementary Information

Supplementary Video
