# Supplementary material for: Hydrogen peroxide dynamics in subcellular compartments of malaria parasites using genetically encoded redox probes
Source: Sci Rep. 2017 Sep 5;7:10449. doi: 10.1038/s41598-017-10093-8 (PMC5585161; doi:10.1038/s41598-017-10093-8)
Supplement: Supplementary file 1 — Supplementary Information [file 41598_2017_10093_MOESM1_ESM.pdf]

Supplementary Information

Hydrogen peroxide dynamics in subcellular compartments of malaria parasites using genetically encoded redox probes

Mahsa Rahbari, Stefan Rahlfs, Jude M. Przyborski, Anna Katharina Schuh, Nicholas H. Hunt, David A. Fidock, Georges E. Grau, Katja Becker

Supplementary Table S1. Effects of antimalarial drugs on the redox ratio of recombinant roGFP2-Orp1 *in vitro.*

Drugs	Increase in fluorescence ratio [a]						Fold change of fluorescence ratio [b]					
	3 min		4 h		24 h		3 min		4 h		24 h	
CTL	0.50 → 0.51		0.50 → 0.72		0.50 → 1.08		1.02		1.44		2.16	
[mM]	1	0.1	1	0.1	1	0.1	1	0.1	1	0.1	1	0.1
AQ	0.41	0.42	0.59	0.66	0.72	1.01	0.80	0.82	0.82	0.92	0.67	0.94
LUM	0.30	0.44	0.43	0.65	0.69	1.05	0.59	0.86	0.60	0.90	0.64	0.97
PQ	0.45	0.49	0.90	0.78	1.28	1.34	0.88	0.96	1.25	1.08	1.19	1.24
ATQ	0.33	0.44	0.49	0.67	0.77	1.08	0.65	0.86	0.68	0.93	0.71	1.00
Rot	0.46	0.48	0.64	0.73	0.98	1.14	0.90	0.94	0.89	1.01	0.91	1.10
2-DG	0.47	0.5	0.70	0.72	1.14	1.08	0.92	0.92	0.97	1.00	1.06	1.00
Comp 1o	0.39	0.43	0.48	0.66	0.71	1.04	0.76	0.76	0.67	0.92	0.66	0.96
ML304	0.45	0.45	0.66	0.68	1.02	1.06	0.88	0.88	0.92	0.94	0.94	0.98

[a] In this column the absolute change in the fluorescence ratio 405/480 nm of isolated recombinant pre-reduced roGFP2-Orp1 after incubation with the antimalarial drugs and redox-active compounds at given concentrations and different time points is shown. Furthermore, the basal ratio 405/480 nm of recombinant roGFP2-Orp1, which served as the starting point for the experiments, is given.

[b] In this column the fold change in the fluorescence ratio 405/480 nm of isolated recombinant roGFP2-Orp1 after incubation with the compounds at given concentrations and time points is shown.

Supplementary Table S2. EC_{50}-values of antimalarial drugs and redox-active compounds on *P. falciparum* NF54-*attB* parasites determined via the SYBR Green assay.

Compound	EC_{50} value	Compound	EC_{50} value
**ART**	5.7 ± 0.9 nM	**LUM**	10.7 ± 1.8 nM
**ATM**	3.4 ± 0.4 nM	**PQ**	8.1 ± 0.2 µM
**ATS**	4.2 ± 0.6 nM	**ATQ**	0.5 nM
**CQ**	6.9 ± 0.8 nM	**Rot**	22.9 ± 6.3 µM
**QN**	66.2 ± 8.2 nM	**2-DG**	6.7 ± 0.6 mM
**MQ**	23.4 ± 2.8 nM	**Compound 1o**	5.6 ± 1.5 nM
**AQ**	3.4 ± 0.2 nM	**ML304**	1.7 µM

Supplementary Table S3. EC_{50}-values of antimalarial drugs and redox-active compounds used for 4 h and 24 h incubation experiments with *P. falciparum* NF54-*attB* parasites.

Compound	EC_{50} value	24 h incubation<br>10 x EC_{50}	4 h incubation<br>100 x EC_{50}
**ART**	6 nM	60 nM	600 nM
**ATM**	3.5 nM	35 nM	350 nM
**ATS**	4 nM	40 nM	400 nM
**CQ**	7 nM	70 nM	700 nM
**QN**	66 nM	660 nM	6.6 µM
**MQ**	23 nM	230 nM	2.3 µM
**AQ**	3.5 nM	35 nM	350 nM
**LUM**	11 nM	110 nM	1.1 µM
**PQ**	1 µM (fixed)	1 µM (fixed)	1 µM (fixed)
**ATQ**	0.5 nM	5 nM	50 nM
**Rot**	10 µM (fixed)	10 µM (fixed)	10 µM (fixed)
**2-DG**	1 mM	10 µM (fixed)	10 µM (fixed)
**Compound 1o**	5 nM	50 nM	500 nM
**ML304**	1.3 µM	1 µM (fixed)	1 µM (fixed)

Supplementary Table S4. Primer sequences for cloning the mitochondrial target sequence citrate synthase (CS) and fusing it to roGFP2-Orp1 (for previous transient transfection experiments with pARL1a+ expression vector), and for cloning the roGFP2-Orp1 (cytosol) construct as well as CS-roGFP2-Orp1 (mitochondrion) construct (→ Mito-roGFP2-Orp1) into the expression vector pDC2-CAM-*attP* for genomic integration in-cell experiments.

**CS**	
OPfCSs	5’-ggGGTACCATGGAAGGAATAAGATACCTATC-3’
OPfCSasN	5’-ggGGTACCTTTCAAAATATTCATAATAACAGATT-3’
**roGFP2-Orp1** (pARL1a+)	
OroGFP2Kpn2s	5’-atatGGTACCAGCAAGGGCGAGGAGCTGTT-3’
OOrp1Xmaas	5’-atatCCCGGGTTATTCCACCTCTTTCAAAAGTTC-3’
**CS-roGFP2-Orp1** (pDC2-CAM-*attP*)	
OCSAvrs	5’-atatCCTAGGATGGAAGGAATAAGATACCTATC-3’
OOrp1Xhoas	5’-atatCTCGAGTTATTCCACCTCTTTCAAAAGTTC-3’
**roGFP2-Orp1** (pDC2-CAM-*attP*)	
OroGFPAvrs	5’-atatCCTAGGATGTGAGCAAGGGCGAGGAG-3’
OOrp1Xhoas	5’-atatCTCGAGTTATTCCACCTCTTTCAAAAGTTC-3’

The restriction sites are underlined.

Supplementary Table S5. Primer sequences for confirmation of genomic integration of constructs in the *P. falciparum* NF54-*attB* strain.

**roGFP2-Orp1/Mito-roGFP2-Orp1**	
cg6 5’	5’-GAAAATATTATTACAAAGGGTGAGG-3’
bsd R	5’-ACGAATTCTTAGCTAATTCGCTTGTAAGA-3’
